# Evaluating blood sampling strategies within the SIREN study: the experience from a large cohort of healthcare workers in the UK

**DOI:** 10.1186/s12874-025-02599-x

**Published:** 2025-07-01

**Authors:** Nipunadi Hettiarachchi, Debbie Blick, Tom Coleman, Ashley Otter, Angela Dunne, Jameel Khawan, Ezra Linley, Michelle J. Cole, SIREN Study Group, Michelle Cairns, Jasmin Islam, Sarah Foulkes, Susan Hopkins, Victoria Hall, Ana Atti

**Affiliations:** 1 Antimicrobial Resistance and Healthcare Associated Infections Division, United Kingdom Health Security Agency (UKHSA), 10 South Colonnade, Canary Wharf, London, E14 4PU UK; 2 Emerging Pathogen Serology, UK , Health Security Agency (UKHSA), Manor Farm Road, Porton, Salisbury SP4 0 JG UK; 3 Vaccine Evaluation Unit (VEU), United Kingdom Health Security Agency (UKHSA), Manchester Royal Infirmary, Oxford Road, Manchester, M139 WL UK

**Keywords:** Evaluation, Blood specimen collection, Phlebotomy, Cohort studies

## Abstract

**Background:**

Delivering research studies that require a large number of samples to monitor specific populations is complex, often resulting in high costs and intricate logistics. We aim to describe the processes for blood sample collection and management and evaluate alternative sampling methods within a large cohort of healthcare workers in the UK (the SIREN study).

**Methods:**

We conducted a process evaluation. First, we described blood sample collection and management across different study periods from June 2020 to March 2024 and how these evolved over time. Secondly, we compared alternative methods of blood sampling: venous phlebotomy (hospital-based) vs. capillary sampling (at-home).

**Results:**

The main challenges with blood sampling within SIREN stemmed from the scale and use of decentralised phlebotomy across 135 hospital sites during the COVID-19 pandemic. We adapted our sampling processes as the study progressed, overcoming most of these challenges. When comparing hospital-based and at-home sampling, overall, return rates of samples taken at home were higher than site- based samples (80% vs 71%, respectively). At-home samples took less time to be returned to UKHSA Laboratory for testing compared to hospital-based samples (median 2 days; interquartile (IQ) 2–3) vs 6 days; IQ 3–8). However, at-home samples were more likely to be considered void (4%) when tested compared to hospital-based samples (0%). Cost for hospital-based sampling was almost 3-times higher than at-home sampling (£34.05 vs £11.50, respectively), although larger sample volumes were obtained via hospital-based sampling when compared to at-home sampling (8 ml vs 600 µl of whole blood).

**Conclusions:**

Sample collection and management in large scale research studies are complex. Our results support at-home blood sampling as an effective and cheaper strategy when compared to hospital-based phlebotomy and therefore should be considered as alternative sampling method for future research.

**Trial registration number:**

ISRCTN11041050—registration date 12/01/2021.

**Supplementary Information:**

The online version contains supplementary material available at 10.1186/s12874-025-02599-x.

## Background

Sample collection is a key element for clinical health research, involving several types of biological specimens depending on the study objectives. Blood samples are among the most common specimens required for surveillance studies, as they are relatively easy to obtain and can provide several insights into diagnosis of diseases, participants’ immune response and medical conditions [1].

The global COVID-19 pandemic required investigating multiple features of an emerging pathogen, including assessing population immunity. To address this, several studies were established and required large numbers of blood samples for immunological testing. However, implementing efficient processes for blood collection at scale during an ongoing pandemic has been challenging [2, 3].

The ideal blood sampling method depends on several aspects, including factors related to the study design, the infrastructure available and participant acceptability [4, 5]. Recently, at-home self-sampling has become more popular and has been applied by clinical studies in a variety of settings, including research on human immunodeficiency virus (HIV), other sexually transmitted infections (STI) and rheumatoid arthritis (RA) [6, 7]. A community-based study in the United Kingdom (UK) was one of the first to use capillary blood samples to evaluate the antibody responses following SARS-CoV-2 vaccination and successfully provided insights into SARS-CoV-2 immune response early in the pandemic [8]. Other studies have demonstrated a strong correlation in SARS-CoV-2 antibody titres between paired plasma samples obtain via phlebotomy and whole blood capillary samples [9].

The SARS-CoV-2 Immunity and Reinfection Evaluation (SIREN) study is a large cohort of healthcare workers in the UK set up in June 2020. The study originally aimed to evaluate whether the presence of antibodies against SARS-CoV-2 would reduce the risk of SARS-CoV-2 reinfection, requiring large scale blood sampling for serological testing [10]. Venous phlebotomy at hospital sites was carried out by trained personnel from the beginning of the study. However, in 2024, at-home capillary sampling was implemented for participants from sites that no longer offered phlebotomy, as an alternative method for sample collection.

Here we describe the evolving methods for blood sample collection across the course of the SIREN study (2020–2024) and evaluate processes and outcomes for venous phlebotomy (hospital-based) and capillary sampling (at-home). This study extends existing knowledge by providing a comparative analysis of real-world implementation of the two distinct blood sampling methods at scale, allowing a head-to-head comparison of feasibility, costs and scalability.

## Methods

### Study design

We conducted a process evaluation of blood sampling within in the SIREN study. The SIREN study protocol is described elsewhere [10].

### Description and review of blood sample collection and management processes over the study period

For the purpose of this analysis, SIREN 1.0 corresponds to the period between June 2020 and March 2023 and SIREN 2.0 corresponds to the period between May 2023 and March 2024. We described and compared the two different study periods in terms of bleeding schedule, sampling methods, sample flows, sample labelling and sample testing. We then reviewed challenges across the periods for the same aspects mentioned above, based on internal reviews and feedback from hospital sites collected in real-time over the duration of the study.

### Evaluation of processes and outcomes for hospital-based and at-home blood sampling

We compared metrics on logistics, sample recovery and costs to evaluate whether there was a superior method for blood sample collection.

#### Sample collection and return

During SIREN 1.0, hospital sites carried out monthly or quarterly venous phlebotomy depending on site capacity. Blood samples were transferred to local laboratories, where they were tested for SARS-CoV-2 antibodies utilising locally validated assays. A serum aliquot from each sample was obtained, labelled, and shipped to UKHSA laboratories via couriers for biobanking and further testing (if required).

In SIREN 2.0, all participants linked to hospital sites were bled at four defined timepoints (September 2023, November 2023, January 2024, and March 2024). Full blood samples were labelled and sent via couriers to a UKHSA laboratory for SARS-CoV-2 antibody testing and biobanking.

During SIREN 2,0, at-home sampling and provided with capillary blood kits (Preventx Limited) including as finger prick devices (BD Microcontainer Contact-Activated lancets) was offered to participants not linked to hospital sites at two time points: January 2024 (Postal bleed 1) and March 2024 (Postal bleed 2). Sample tubes were pre-identified with correspondent study sample IDs and returned to UKHSA Porton Down by participants using the pre-paid tracked 24 h postal service for testing, following UN3373 regulations [11].

#### Data collection

For serum samples collected at hospital sites within SIREN 1.0, study participant IDs, study sample IDs and collection dates were obtained from the electronic manifest shared by the site with the UKHSA Sero-epidemiology Unit team. These data were checked against the samples received at the UKHSA laboratories, errors investigated and corrected, and the data uploaded to a custom database built for the SIREN study.

For blood samples collected at hospital sites within SIREN 2.0, study sample IDs and collection dates were obtained from the electronic manifest shared with the UKHSA SIREN team, which was uploaded into the UKHSA Laboratory Information Management System (LIMS) and checked against the samples received at the UKHSA laboratories.

For at-home blood sampling, participants were asked to complete a referral form including their study sample ID and collection dates. These were entered manually into LIMS by the laboratory team upon receipt of samples.

The UKHSA LIMS also captured information on sample receipt date and antibody testing results, which were linked to participants via study sample IDs.

#### Data analysis

Sample return rates were calculated by using the number of returned samples divided by the number of expected samples for a specific study period. The number of returned samples was retrieved from LIMS, based on their arrival and logging of associated data in LIMS.

The expected number of samples was calculated for each hospital site based on the number of participants recruited who had not withdrawn at time of the scheduled bleeds. The timeliness of sample return was determined by calculating the median days between sample collection and laboratory receipt dates.

We described the rates of void samples for each sampling method by using the number of void samples divided by the total number of samples returned. Samples could be considered void due to insufficient volume for processing, clotted, or if they fail the assay’s quality control criteria.

The cost per sample for phlebotomy was calculated based on information from the financial reports provided by hospital sites. For at-home sampling, we used costs associated with procuring and assembling sample kits and inbound shipment. Outbound shipments, either from hospital sites to UKHSA or from participants to UKHSA, were calculated for both methods.

Sample volumes were provided based on the requirements described at the study protocol for hospital-based sampling (up to 8 mL). For at-home sampling, maximum volumes were obtained from the manufacture of capillary blood kits utilised in this study (up to 600uL).

## Results

### Description and review of blood sample collection and management processes over the study period

**Fig. 1:**
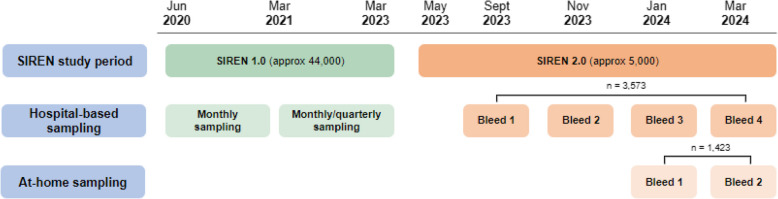
Description of study periods and blood sample collection throughout the SIREN study

Over 44,000 participants joined the SIREN study between June 2020 and March 2021 from 135 hospital sites across the UK, who underwent frequent PCR and serological testing locally. They were initially followed-up for 12-months and offered two subsequent opportunities to extend their participation in the study until March 2023 (SIREN 1.0). From May 2023, a subset of 6,047 participants from the original cohort joined another phase of the study, named as SIREN 2.0, and resumed PCR sampling at home (May 2023-March 2024) with phlebotomy and subsequent serological testing at hospital sites (September 2023-March 2024). At-home blood sampling was established between January and March 2024 for a subset of SIREN 2.0 participants whose hospital sites were not offering serological testing (Fig. 1). A comparison of the main processes related to blood sample collection and management for each study period is captured in Table 1.

**Table 1:** Comparing different processes related to sample collection and management by study period

	**SIREN 1.0**	**SIREN 2.0**	
		**Hospital-based sampling**	**At-home sampling**
Bleeding schedule	Monthly bleeds from June 2020 to March 2021 for all hospital sites. From March 2021 onwards, hospital sites could choose to perform quarterly bleeds instead of monthly Different bleeding schedules for each site and, within a site, different bleeding schedule for each participant	All hospital sites and participants followed the same schedule for bleeds, every two months (September 2023, November 2023, January 2024, and March 2024)	Two pre-defined time points for sample collection (January and March 2024)
Blood sampling and sample flows	Blood samples were collected by phlebotomy/research teams at the hospital sites Local laboratories would extract sera from whole blood samples and send an aliquot to UKHSA laboratory	Blood samples were collected by phlebotomy/research teams at hospital sites Whole blood samples were centrifuged and shipped to UKHSA Porton Down	Participants were provided with a capillary sampling blood kits via post
Sample labelling	Sample labels for all participants and bleeds provided at the beginning of the study period	Sample labels provided at 4 time-points for the immediately upcoming bleed	Capillary sampling kit included a pre-labelled blood tube
Sample shipping	Electronic manifests should capture all samples included in the physical shipment and be sent to UKHSA team at least 2 days prior to shipment Monthly shipments to UKHSA laboratory	Electronic manifests should capture all samples included in the physical shipment and be sent to UKHSA team at least 2 days prior to shipment Samples should be shipped to UKHSA laboratory within 7 days after collection	Whole blood sampled were sent directly to UKHSA laboratory by participants using a pre-paid postal service, as soon as the sample was taken
Sample processing and testing	Samples tested for SARS-CoV-2 serology at hospital sites, utilising locally validated assays If further testing was required, serum samples were shipped from SEU to UKHSA Porton Down	All testing was carried out at UKHSA Porton Down	All testing was carried out at UKHSA Porton Down

Multiple issues were identified in SIREN 1.0 across all aspects analysed (Table 2). Managing different bleeding schedules for each site and participants required considerable staff capacity, both from research and phlebotomy teams. Given the large number of participants per site and frequency of bleeds, several samples were incorrectly labelled or did not match electronic records, which delayed sample shipment and generated a large volume of sample discrepancies. Having antibody testing performed at different laboratories utilising different assays was also problematic and often required confirmatory testing at a UKHSA laboratory for subsequent specific analyses.

**Table 2:** Challenges related to blood sample collection and management throughout the SIREN study

	**SIREN 1.0**	**SIREN 2.0**	
		**Hospital-based sampling**	**At-home sampling**
Bleeding schedule	Each site and participant had their own bleeding schedules to manage, creating considerable staffing demands for phlebotomy clinics and research teams	No issues reported	No issues reported
Blood sampling and sample flows	Phlebotomy clinics required skilled staff and considerable resources, which were costly to the study Samples required aliquoting and processing at local laboratories, which was time and resource consuming Hospital sites were not shipping samples frequently enough, delaying analyses using antibody data	Phlebotomy clinics required skilled staff and considerable resources, which were costly to the study	Few participants reported issues with capillary blood collection, mainly related to not being familiar with finger prick testing
Sample labelling	At the beginning of the study, pre-defined sample ID labels were provided to sites based on an estimated number of participants and bleeds There were delays in sending out the first batches of labels to certain hospital sites due to logistical issues, which led to some sites having to manually write sample IDs to identify samples If participants missed visits, this would create leftover labels that could mistakenly be used for future bleeds Initially, extra labels could be ordered via email, which may have increased errors in label printing and shipment. A label request form for ad hoc label requests was further implemented Delays on delivering additional sample ID labels in time for bleeds during the first study extension resulted in some hospital sites printing their own labels or handwriting labels, leading to transcription errors	Minimal issues with sample mislabeling	No issues reported
Sample shipping	Several shipments received did not match the electronic manifest, requiring in-depth investigation to be resolved and delaying samples getting recorded into the LIMS The electronic manifest was not always sent prior to the physical shipment, which impacted on the ability of managing large shipments in a timely manner Electronic manifests were not completed utilising a barcode reader, which lead to transcription errors	A small number of shipments received where samples did not match the electronic manifest, requiring in-depth investigation to be resolved and delaying samples getting recorded into the LIMS The electronic manifest was not always sent prior to sending the physical shipment, which impacted on the ability managing large shipments in a timely manner Challenges in setting up couriers for sample collection at some hospital sites	Minimal delays on returning samples to the laboratory, only delays associated to postage strikes
Sample processing and testing	Hospital sites conducted different SARS-CoV-2 serological assays. This meant the specificity and sensitivity of these results were not uniform and consistent throughout the study If additional testing was required at UKHSA, samples would have to be transported to a different laboratory, impacting on delays in testing	No issues reported	A few samples were classified as void Due to insufficient volume or due to the blood sample being clotted, samples can fail the assay’s quality control criteria

Fewer challenges were encountered in SIREN 2.0, demonstrating quality improvement over time. SIREN 2.0 hospital-based sampling had a lower number of sample discrepancies or mislabelling than SIREN 1.0. However, some sites in SIREN 2.0 had issues with setting up couriers for sample shipment, which was more time-sensitive than in SIREN 1.0 due to the shipment of whole blood rather than serum. The use of centralised antibody testing at UKHSA laboratories reduced costs and sample logistics at hospital sites. For SIREN 2.0 at-home sampling, most of the remaining issues related to hospital-based sampling were overcome; for example, samples being directly sent to the UKHSA laboratory by participants optimised sample return and pre-labelling minimised any sample discrepancies. Nonetheless, some participants reported challenges with the capillary sampling method and some samples had insufficient volumes for testing (Table 2).

### Evaluation of processes and outcomes for hospital-based and at-home blood sampling

In total, 6,047 SIREN participants consented to join SIREN 2.0. Of those, 3,624 participants underwent hospital-based sampling, whereas 1,423 participants joined at-home sampling.

For hospital-based sampling, sample return rates varied by bleed timepoint from 61 to 77% based on expected numbers of samples. For at-home sampling, the average return rate was higher (80%) (Table 3).

**Table 3:** Return rates from SIREN 2.0 hospital-based and at-home sampling. Numbers of expected samples excludes anyone who had withdrew from the study at time of bleeds

	**SIREN 2.0**					
	**Hospital-based sampling**				**At-home sampling**	
	**Bleed 1**	**Bleed 2**	**Bleed 3**	**Bleed 4**	**Postal bleed 1**	**Postal bleed 2**
**Expected samples (n)**	3,624	3,557	3,488	3,459	1,423	1,411
**Returned samples (n)**	2,735	2,564	2,424	2,081	1,180	1,090
**Return rates (%)**	75%	73%	70%	61%	83%	77%
**Average return rate per pathway (mean %)**	70%				80%	

When comparing the time between sample collection and return to the UKHSA laboratory, samples taken at hospitals took a median of 6 days (IQR 3–8) to be returned whereas samples taken at-home were returned within a median of 2 days (IQR 2–3) (Table 4).

**Table 4:** Timeliness of serology samples received at UKHSA Laboratory from SIREN 2.0 hospital-based and at-home sampling

	**SIREN 2.0**					
	**Hospital-based sampling**				**At-home sampling**	
	**Bleed 1 (** ***n*** ** = 2,735)**	**Bleed 2 (** ***n*** ** = 2,564)**	**Bleed 3 (** ***n*** ** = 2,424)**	**Bleed 4 (** ***n*** ** = 2,081)**	**Postal bleed 1 (** ***n*** ** = 1,180)**	**Postal bleed 2 (** ***n*** ** = 1,090)**
**Number of days for sample return per bleed (median (p50); IQR)**	7 (5–8)	6 (3–8)	5 (3–7)	6 (3–7)	2 (2–3)	2 (2–3)
**Overall number of days for sample return per pathway (median; IQR)**	6 (IQR 3–8)				2 (IQR 2–3)	

Overall, hospital-based samples were less likely to be considered void (0%) compared to at-home sampling (4%) (Table 5). Most frequent reason for void samples from at-home sampling was due to insufficient volume.

**Table 5:** Proportion of void samples from SIREN 2.0 hospital based and at-home sampling

	**SIREN 2.0**					
	**Hospital-based sampling**				**At-home sampling**	
	**Bleed 1 (** ***n*** ** = 2,735)**	**Bleed 2 (** ***n*** ** = 2,564)**	**Bleed 3 (** ***n*** ** = 2,424)**	**Bleed 4 (** ***n*** ** = 2,081)**	**Postal bleed 1 (** ***n*** ** = 1,180)**	**Postal bleed 2 (** ***n*** ** = 1,090)**
**Void samples (n; %)**	1 (0%)	0 (0%)	0 (0%)	0 (0%)	66 (6%)	19 (2%)
**Overall number of void samples per pathway (mean %)**	0%				4%	

Regarding costs, hospital-based sampling was approximately 3-times more expensive compared to at-home sampling (Table 6), excluding consumable costs for hospital-based sampling.

**Table 6:** Estimation of overall cost per sample from SIREN 2.0 hospital-based and at-home sampling. *Shipping charges include both outbound and return mailing costs for at-home sampling.**Phlebotomy charge excludes consumables, given those were covered by the hospital sites

Study stage	SIREN 2.0	
	**Hospital-based sampling**	**At-home sampling**
Shipping charges	£1.11	£6.00*
Serology/Phlebotomy charge**	£16.49	N/A
Participant admin per month per participant	£7.65	N/A
Nurse time per month per participant	£8.80	N/A
Capillary sampling kit charges	N/A	£5.50
**Total**	**£34.05**	**£11.50**

When considering sample volumes, hospital-based sampling offered larger volumes of blood via phlebotomy. As per protocol, during SIREN 1.0, at least 2 mL of sera should be obtained from participants from each bleed. For SIREN 2.0, 8 mL of whole blood samples were expected to be shipped to UKHSA Porton Down for testing for each bleed (ensuring a minimum of 2 mL of sera per bleed). In contrast, capillary blood sampling yields very small volumes; in our study, the maximum volume of blood obtained via at-home finger-prick sampling was 600 μL.

## Discussion

In this study, we described the different processes for blood collection within the SIREN study and how those evolved as the study progressed.

Continuous improvement cycles are key to deliver large scale responsive research, and, although for the purpose of this analysis we have focused on improvements made between SIREN 1.0 and SIREN 2.0, there have been iterative improvements throughout the study.. Our option to utilise a decentralised approach for sample collection and testing in SIREN 1.0 has proved to be complex, requiring extensive logistical arrangements and research site’s local capability. Troubleshooting by UKHSA and the study sites to resolve sample discrepancies was a resource heavy activity. However, by collecting feedback and consistently reviewing processes for sample collection and management, we were able to overcome these challenges in SIREN 2.0, e.g. by standardising bleeding schedules and having centralised testing at UKHSA Laboratories, as opposed to local testing on a range of assays. Some studies have explored the concept of home visits by phlebotomists; however, this is impractical for large scale studies [12].

These processes were further improved by implementing at-home sampling. When comparing hospital-based and at-home sampling, we have demonstrated that self-blood collection was superior in terms of logistics, sample return and costs. Lower return rates for samples collected at hospitals may be justified by the need of participants to attend phlebotomy clinics for blood collection, relying on participant’s and clinics’ availability, which may have contributed to missed appointments. Most hospital opted to batch and ship their samples to UKHSA on a weekly basis to ease the burden with logistics, delaying the return of individual samples. Additionally, supply shortages, although limited to a few hospitals during a one-month period in 2023, also contributed to lower return rates and longer return-time for samples collected at hospitals compared to at-home sampling.

Our findings are supported by previous studies, that have found that capillary blood sampling was an accurate and advantageous method for blood sample collection for assessing SARS-CoV-2 antibodies and other serological markers [13–15]. Furthermore, in the context of an outbreak of a new or emerging pathogen, at-home sampling minimises the risk of disease transmission between individuals [16].

Despite low numbers, at-home sampling provided a higher rate of voided samples upon testing when compared to phlebotomy, generally due to insufficient volumes of samples, which could have implications for specific analyses. It should be recognised that at-home sampling can only extract low sample volumes, that could become insufficient for testing if the correct instructions for blood collection are not followed and limiting the options of running additional testing or biobanking samples. [17] In addition, at-home sampling would not be suitable for specific analysis that require rapid sample processing and where sample stability might be a concern, although it has been demonstrated that, for antibody testing against different viruses, whole blood samples could be kept in room temperature for up to 6 days without affecting results [18]. These are key limitations of self-sampling methods and should be considered when designing new research studies, to ensure the study requirements are fulfilled.

Another important aspect when considering different methods for blood sampling is participants’ experience. Previous studies have demonstrated self-collection methods to be highly accepted by participants, [5, 6, 14] although some reported it to be uncomfortable or painful [19]. We collected SIREN participant’s feedback on hospital-based and at-home sampling and preliminary analysis found that although both methods were acceptable, at-home sampling was preferable compared to hospital-based (55% vs 23%, respectively). This will be described in more details in a separate analysis.

This analysis has limitations. First, as a fast-paced, large-scale study, it was not possible to systematically evaluate some of the improvement strategies introduced in the initial phases of the study. Still, our intention in this manuscript was also to describe the challenges and learning from the study’s early stages, some of which are quite specific to delivering research in a pandemic. Second, the absence of structured tools for obtaining feedback from hospital sites throughout the study may have limited our observations on challenges faced by them regarding sample collection and processing; nonetheless, our continued engagement via frequent meetings and open communication channels allowed us to capture the main constrains. Third, our evaluation comparing hospital-based and at-home sampling did not include post-analytical metrics related to the performance of the assay, as quality indicators were not available at the time of this analysis. However, previous studies have shown high rates of concordant SARS-CoV-2 antibody results between the two different methods [5, 7, 9, 15].

Overall, we have demonstrated capillary blood collection is advantageous compared to venous phlebotomy, mindful of limited sample volumes, with additional benefits in terms of logistical arrangements and convenience for sampling.

## Conclusions

Large scale research studies that require the collection of blood samples can face challenges related to sample collection and management. Overall, at-home blood sampling is a valuable tool for research testing, providing cost-effectiveness and convenience for participants.

## Supplementary Information

Supplementary Material 1: A complete list of the SIREN Study Group investigators.

## Data Availability

Anonymised datasets used and/or analysed during the current study are available from the corresponding author on reasonable request.
